# Kidney Diseases

**Published:** 1894-07-07

**Authors:** 


### Medical Progress.

### KIDNEY DISEASES.

The discussion on the diet in Bright's disease has nearly ceased for a time. Aufrecht[1] in discussing the treatment of acute nephritis, advises that it should be most carefully watched for in the course of fevers and other diseases. As soon as albumen is found the patient should be kept in bed and fed on non-nitrogenous food to lessen the work of the kidney. If the quantity of urine becomes lessened, it is because the tubules are blocked with casts. These can be removed by alkaline saline water, such as Wildungen. In uræmia he uses a warm wet pack, but fears the risks of pilocarpin, and generally gives up entirely all diuretics.

The treatment of uræmia and its causes have of late occupied general attention. Beverley Robinson[2] advocates the use of large saline enemata and venesection or cupping. Digitalis in some cases may increase the contraction of the vessels of the kidney, purgation and diaphoresis remove but small quantities of the poisonous excretion and diminish the flow through the kidneys. On the other hand, high enemata stimulate the kidneys, dilute the poisons by being absorbed into the blood, and the portion of them which comes away from the bowel brings with it many fermentation products. Venesection in strong patients and cupping in weaker ones will often save life when other remedies fail. For convulsions, other remedies such as chloroform or chloral are often of value, a strict diet and intestinal antisepsis are of great importance. While he refers to the risks, he acknowledges the occasional benefits of pilocarpine, and where neither venesection, high enemata, nor saline intravenous injections are employed he finds that hypodermic injections of sterilised saline solutions are useful, especially in the uræmia which comes on after acute and exhausting diseases. For renal dyspnœa he relied on counter-irritation over the lungs, the use of nitrites and oxygen. In the discussion which followed this paper Dr. Delafield laid stress on bringing about arterial dilatation by any means where there was high tension, and on the wet pack for cases of true chronic uræmia. Roosevelt argued the importance of venesection and of hypodermic saline injections, while others advocated for convulsions the use of urethan.

Three or four hundred grains could be given in 24 hours; it was found to be harmless, and controlled convulsions when everything else failed.

C. A. Herter[3] gave a critical account of the existing theories of uræmia, and concluded his paper before the New York Neurological Society by deciding that the gastro-intestinal symptoms were due to urea poisoning. Some cases of uræmia, however, were probably due to infective processes; some to the accumulation of urea and extractives in the blood. The importance of urea in the gastro-intestinal class is shown (1) by the excess of urea in the blood; (2) by the production of similar symptoms when urea was injected; (3) by the occurrence of urea in the fæces of these patients; (4) by the absence of toxicity in the urine or elevation of temperature such as results from septic conditions; (5) by the presence of gastro-intestinal congestion, such as can be caused by the injection of urea. This explanation does not apply to other types of uræmia.

Professor N. S. Davis[4] warns us to watch for the initial symptoms, to keep the patient in bed, and give only non-nitrogenous food without even milk for a time. Among these symptoms are headache, diarrhœa, asthma, gastric disturbances and diarrhœa, occasional blindness, insomnia, buzzing in the ears, numbness and formication. Large doses of carbonate of iron often relieve these symptoms. Though salicylates favour the elimination of urea, they have little effect in preventing uræmia. He employs ordinary diaphoretics, diuretics, and purgatives with iron, and praises lactose in some instances. Profuse sweating in œdematous persons may bring on acute uræmia.

Dr. Lendon,[5] reporting a case where one kidney had apparently been affected long before the other, suggests that acute mononephritis may explain instances of acute Bright's disease when occurring without dropsy.

Coltman[6] contributes from Pekin, China, three cases of nephritis with dropsy and albuminuria treated most successfully by drop doses of icthyol.

Curschman,[7] for the removal of anasarca, uses a large flat Southey's needle, to which the cannula fits closely. This needle is introduced under the skin at an acute angle, and with antiseptic precautions. When the fluid does not flow freely he fastens a glass cup and tube over some small incisions, and allows the fluid to drain off through it.

At a discussion which took place at the Royal Academy of Medicine in Dublin, free purgation, leeching, and the use of diuretin were advocated in acute nephritis, but opinions were much divided as to the use of the latter.[8]

The subject of uræmic hemiplegia has been carefully discussed by R. W. Wilcox.[9] The pathology is referred to serous œdema of the brain, like the fugitive œdema of the eyelids, and possibly in some cases to the action of toxins on the nerve centres. The previous symptoms vertigo, albuminuria, localised œdemas, low specific gravity, and hyaline casts, with gastric disturbances, are important in making a diagnosis. There is sudden hemiplegia, with or without unconsciousness, followed after a time by complete recovery or change of limbs affected. Bleeding or ordinary renal treatment is recommended.

The mode in which changes are produced in the heart and vessels in renal disease is referred[10] by Dr. G. A. Gibson to peripheral resistance, caused by the presence of waste products, and possibly by the increased friction of the blood, which results when the specific gravity of the blood is diminished and the leucocytes mix with the red corpuscles. This would explain the high tension often found in anæmia. However, Dr. G. A. Gibson quotes from Dunlop that a mixture of urine with blood stimulates the frog's heart to very powerful contractions, so that cardiac hypertrophy may not altogether depend on increased peripheral resistance. It is the increased exertion which in either case produces the hypertrophy, and the relation of the thickness of the ventricular walls to the work they have to do is shown by comparison of the fœtal heart with that of a pregnant woman. In the latter the left ventricle is greatly hypertrophied, since it has to drive forward blood for excreting the waste products of mother and child. In the former both sides of the heart are equal, as is the work done by each.

The connection between disease of the kidney and heart disease is shown in (*a*) interstitial nephritis, where the heart hypertrophy may be either secondary to the kidney disease, or a common result of endarteritis. Tyson[11] advises the elimination of nitrogenous food from the dietary, and gives iodide of potash and the nitrites (*b*) Mitral regurgitation may cause cyanotic induration of the kidney. Here he employs a restricted milk diet, with large doses of digitalis, sparteine, or strophanthus. (*c*) Embolic infarction of the kidneys from vegetations on a valve of the heart, or from a thrombus, calls for no special treatment. Sudden pain and hæmorrhage from the kidney, with previously-existing heart disease, are the only symptoms.

Renal inadequacy, or "the state of the kidney in which it is unable, without a material diminution of quantity to produce a urine containing the average amount of solids and a specific gravity greater than 1,014," is shown by Gifford to closely resemble myxœdema on the one hand, and certain forms of Bright's disease without albuminuria on the other. Constant liability to colds, bad appetite, inability to take much fluid or stimulants, or to undergo fatigue or operations are among the symptoms found. The administration of thyroid extracts in some of these cases results in an increased excretion of urea and general improvement. An account of several cases with reference to others treated by Ord, White, and Napier is contributed by Gifford to the *Lancet*.

In the treatment of renal insufficiency Rochester[13] protests against the use of stimulating diuretics or digitalis, unless after purgation has relieved the venous congestion. Attention to diet, and regular exercise or massage, inhalation of oxygen, the use of distilled or alkaline water and iron are important. In place of medicines to act on the kidney, he relies on hot baths and massage for stimulating the skin; purgatives and copious enemata for acting on the mucous membrane of the bowels.

D. D. Stewart[14] discusses six instances of non-albuminuric disease of kidneys other than typical fibroid. Three were young adults and three were in middle life. All presented headache, vertigo, high tension pulse, pain in loins, diminution of urine and of urea, the presence of casts and of renal epithelia, the absence of albumen and of dropsy. Eye symptoms were noted in several cases. The pathology is not clear, but Stewart believes that some degree of fibroid change is present in the kidney, with some involvement of the tubules and glomeruli.

General dropsy has been found by several observers to follow prolonged retention of urine in the bladder, and Lepine's experiments on dogs show that increased pressure of urine, if constant and invariable, greatly decreases its amount without inducing albuminuria.[15] A short method for the estimation of uric acid is given by Arthaud and Butte.[16] Precipitate phosphates by an excess of sodium carbonate and filter. Take 50 c.c. of the filtrate and triturate with copper hyposulphate. The filtrate should be warmed before testing, and the trituration continued until all the uric acid is precipitated, or the end point can be ascertained by adding a little of the filtered mixture to a 10 per cent. solution of sodium xanthogenate, which gives a yellow precipitate with copper. Ten c.c. of the standard solution is equal to 0·02 gramme uric acid. This copper solution is made by taking (*a*) copper sulphate 14·84 grm., tartaric acid a pinch, water to a litre; (*b*) sod. hyposulphate 80 grm., Rochelle salt 160 grm., carbolic acid a few drops, water to a litre. Mix one part of *a* with four of *b* before using.

Dr. L. Dickenson has recently[17] shown three cases of transient hæmoglobinuria from muscular exertion with no serious symptoms. The attacks followed athletic exercises. Such cases are akin to functional albuminuria, and imply some delicacy of the red corpuscles. In these instances the blood seemed healthy, but the corpuscles were destroyed by some product of the muscular exertion, probably carbonic acid.

[1] Therap. Monatsh, Oct., 1893. [2] Med. Rec., March 17, 1894. [3] Med. Week., April 3, 1894. [4] Int. Clinics, Vol. III., 1893. [5] Lancet, April 7, 1894. [6] Clin. Jour., April 11, 1894. [7] Med. Chron., April, 1894. [8] Lancet, April 7, 1894. [9] Am. Jour. Med. Sc., May, 1893. [10] Int. Clin., Vol. III., 1893. [11] Int. Clin., Vol. IV., 1893. [12] Lancet, March 31, 1894. [13] Med. Rec., May 19, 1894. [14] Amer. Jour. Med. Sc., Dec., 1893. [15] Med. Week., April 16, 1894. [16] Chem. and Druggist, March 31, 1894. [17] Med. Week., May 18, 1894.

